# Metallothionein-like 5 expression is correlated with poor prognosis and promotes proliferation of cervical squamous cell carcinoma

**DOI:** 10.1080/21655979.2022.2036901

**Published:** 2022-03-07

**Authors:** Yi Huang, Qin Wu, Xiaoqing Tan

**Affiliations:** Department of Obstetrics and Gynecology, Chongqing General Hospital, Chongqing, China

**Keywords:** Cervical squamous cell carcinoma, metallothionein-like 5, prognostic biomarker, proliferation

## Abstract

Cervical cancer represents one of the most important female genital cancers. Cervical squamous cell carcinoma (CESC) accounts for about 90% of all cervical malignancies and the prognosis are unsatisfied. Here we aimed to investigate the clinical relevance of metallothionein-like 5 (MTL5), a novel metallothionein-like protein, in CESC. RT-qPCR and immunohistochemistry staining showed that MTL5 was upregulated in CESC tissues than nontumorous cervix tissues, which is consistent with the data from TCGA database. Kaplan–Meier survival analysis revealed that higher MTL5 can help predict worse prognosis. In addition, Cox hazard regression analysis verified an independent predictive role of MTL5 in CESC. To further investigate the involvement of MTL5 in CESC, we conducted knockdown experiments in two CESC cell lines. As a result, silencing MTL5g significantly inhibited proliferation of CESC cells. Finally, we validated that silencing MTL5 can suppress CESC tumor growth in vivo using the mice subcutaneous xenografts model. Taken together, higher MTL5 indicates worse survival of CESC after surgical resection. Targeting MTL5 represents a potential therapy of CESC by inhibiting tumor growth, which deserves further investigations.

## Graphical AbstractMTL5 expression level is significantly upregulated in CESC tissues (A) and is negatively correlated with prognosis of CESC patients (B), which may function by enhancing CESC growth as revealed by in vivo xenograft model (C)

**Figure:**
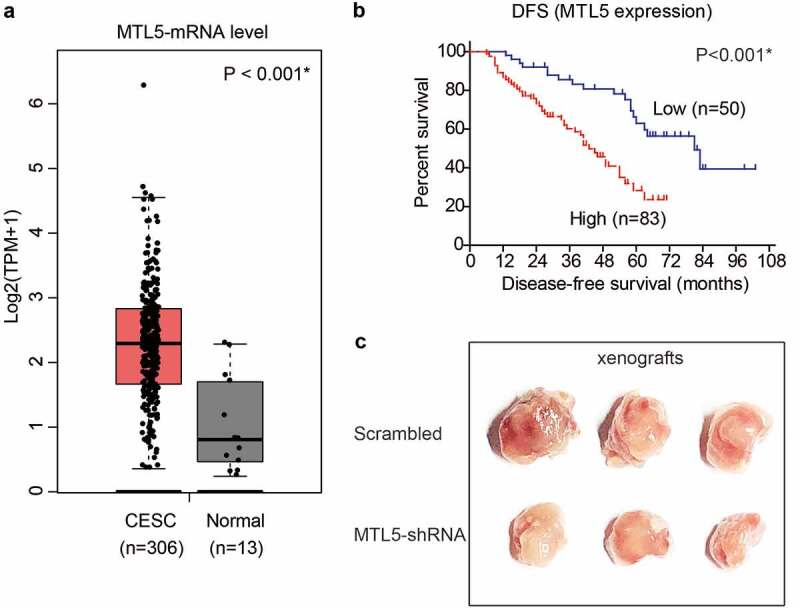

## Introduction

Metallothionein refers to a family of cysteine-rich protein with low molecular weight. The high constituent of cysteine ensures its capacity to bind heavy metals, including zinc, copper, cadmium, silver, etc. [1]. Therefore, metallothionein was initially identified to play functions in the protection against metal toxicity, as well as in zinc and copper regulation [2–4]. Expression of metallothionein can also be upregulated by oxidative stress to protect the cells against cytotoxicity and DNA damage [5]. Of note, metallothionein plays a critical role in regulating protein transcription due to its capacity to bind zinc, which is a key element for activating transcription factors such as zinc figure proteins. Therefore, dysregulated metallothionein expression or function may result in carcinogenesis [6].

Metallothionein-like 5 (MTL5), also named as TESMIN (Testis Expressed Metallothionein Like Protein), was firstly isolated by Sugihara et al. in 1999, which is a cysteine-rich protein containing two metallothionein-like motifs [7]. Initially, MTL5 was thought to be uniquely expressed in spermatocytes and participates in male germ cell differentiation [8]. However, lower but detectable expression of MTL5 in other tissues were also reported in the past decades, such as ovary, breast, parathyroid gland, etc. [9]. Dysregulated MTL5 had also been recognized to play potential roles in lung cancer. Genome-wide gene expression analyses revealed that MTL5 was closely associated with the molecular pathogenesis of lung adenocarcinoma (LUAD) [10]. In addition, higher MTL5 expression in LUAD can serve as an independent predictive factor for overall survival of LUAD patients in TCGA database [10]. Later, it was reported that MTL5 expressed higher in lung cancer cells compared to control cells and its expression may help predict prognosis, suggesting its role in carcinogenesis [11]. However, till now, lung cancer is the only malignancy that has been reported to be associated with MTL5. The expression and function of MTL5 in other malignancies remain unknown, which restrained its further investigation and application in clinical practice

Here we aimed to explore the mRNA and protein expression of MTL5 in cervical squamous cell carcinoma (CESC) by using our retrospective cohort as well as online data mining. Besides, we investigated the clinical relevance of MTL5 in CESC for the first time, which determined its significance on predicting CESC patients’ survival, highlighting its tumor-related role in human malignancies. Finally, we validated that MTL5 can enhance CESC growth through both in vitro and in vivo strategies.

## Materials and methods

### Patients and clinical specimens

Paired tumor tissues and adjacent cervical tissues of 17 CESC patients were collected and fresh-frozen in liquid nitrogen in the Chongqing General Hospital, which were used for mRNA analyses. Another 133 formalin-fixed paraffin-embedded (FFPE) CESC tissue samples were also collected with intact survival information from Chongqing General Hospital. As for the 133 cases, all patients underwent R0 surgical resection and staged as FIGO (International Federation of Obstetrics and Gynecology) stage I–II. All tissue samples had been pathologically examined by the Department of Pathology of Chongqing General Hospital. This study had acquired the approval of the Ethics Committee of the Chongqing General Hospital. Written informed consent was obtained from each patient.

### Real-time quantitative reverse transcription PCR (RT-QPCR)

The total RNA from tissue samples was extracted with a TRIzol reagent extraction kit (Invitrogen). The reverse transcription was performed with the SuperScript First-Strand Synthesis system (Invitrogen). Quantitative assay of gene expressions was performed by an SYBR Green qPCR Kit and an ABI 7500 real-time PCR system (Applied Biosystems). The gene expressions were normalized to the GAPDH and calculated using the 2-ΔΔCT method. The specific primer sequences were designed and synthesized by GenePharma (Shanghai, China).

### Immunohistochemical (IHC) staining

The FFPE tissues were sliced into 5-µm slices and subjected to IHC staining according to the standard procedures [12]. Primary antibody targeting MTL5 was purchased from Novus Biologicals (Cat. #NBP2-13,624). The stained slides were scored for the intensity of staining (0 to 3) and the percentage of stained cells, with scores of 0 (0%), 1 (1% to 25%), 2 (26% to 49%), 3 (50% to 75%), and 4 (76%–100%). IHC score (0 to 12) was defined as the product of the staining intensity and percentage of stained cells. MTL5 expression was judged as high-expression level when the IHC score ≥5. All IHC results were evaluated by two experienced pathologists who were blinded to the condition of the patients.

### Cell culture

Human CESC C33A and SiHa cell lines were purchased from ATCC (MD, USA) and cultured in DMEM (Dulbecco’s Modified Eagle Medium) containing 10% FBS (fetal bovine serum) and 1% P/S (penicillin/streptomycin) at 5% CO2 at 37°C. Lentivirus-MTL5-shRNAs (MTL5-sh#1, MTL5-sh#2) and negative vectors containing scrambled shRNA (Scram-sh) were synthesized by GenePharma. For the transfection, cultured cells (2 × 10^5^) were seeded in a 6-well plate for 12 h for attachment. Then, cells were transfected with the plasmids mentioned above using Lipofectamine™ 2000 (Thermo Fisher Scientific, USA) according to the manufacturer’s instruction. The transfection efficiency was determined by Western blotting assay [13].

### Western blotting (WB)

Forty-eight hours after transfection, cells were collected and washed with PBS. Total proteins were extracted from the lysate and fractionated using sodium dodecyl sulfate polyacrylamide gel electrophoresis (SDS-PAGE). The proteins were transferred onto a nitrocellulose membrane for WB analysis according to the standard procedures [14]. GAPDH was used as the internal control.

### CCK‐8 assay

Transfected cells (2 × 10^5^) were seeded in a 96‐well plate (Corning, USA) and cultured for 6 h, 24 h, 48 h, 72 h, and 96 h, respectively. At each time point, 10 μL from CCK‐8 kit reagent (Dojindo, Japan) was added to each well and incubated for 1 h. The cell proliferation was measured using the microplate reader (Bio-Rad, USA) at 450 nm absorbance [15].

### Tumor xenograft models

Female BCLB/nude mice were obtained from Shanghai Laboratory Animal Center (Shanghai, China). The mice were housed in a standard laboratory condition (22°C with 60% humidity). All animal experiments and procedures were performed in accordance with the guidelines of animal welfare of Chongqing General Hospital. The animal experiments were approved and supervised by the Ethics Committee of the Chongqing General Hospital.

Transfected cells were diluted and subcutaneously injected under the skin of the nude mice. Then, the tumorigenesis and tumor growth of the mice were monitored and recorded for four weeks [16]. The volumes of xenografts were calculated with the formula: V = length × width^2^ × π/6. After then, all mice were sacrificed, and xenografts were isolated.

### Statistics

All statistical analyses were performed using SPSS version 18.0 (IBM, NY, USA). Data were presented as mean ± standard deviation (SD). Statistical differences between the groups were compared using Student’s t-tests. Disease-free survival (DFS) was defined as the survival period from the time of surgical resection to tumor recurrence or death. To explore the associations between DFS and clinicopathological characteristics, Kaplan–Meier survival analyses were performed. Two-sided P < 0.05 was considered to be statistically significant.

## Results

We hypothesized that MTL5 may participate in the progression of CESC, therefore, we tested the expression profile of MTL5 in CESC from TCGA database as well as a retrospective cohort in our medical center (n = 133). As a result, we found that MTL5 was higher expressed in certain CESC tissues, which was correlated with aggressive tumor characteristics and worse prognosis. Besides, knockdown of MTL5 resulted in attenuated CESC cell proliferation capacity. Finally, xenograft assays validated the tumor-promoting role of MTL5 in CESC.

### Patients’ information

The clinicopathological characteristics of the 133 CESC cases were recorded including patients’ age, horizontal diffusion diameter, stromal invasion depth, vagina invasion, parametrial invasion, lymphovascular invasion, pathological differentiation grade, lymph node metastasis, FIGO stages, and DFS time (Table 1). In this cohort, 62 cases were diagnosed before 45 years old, while the others were diagnosed at or older than 45 years old. Nighty cases exhibited the horizontal diffusion diameter as less than 4.0 cm, and the other 43 cases larger than or equal to 4.0 cm. Among them, 78 patients were characterized as stromal invasion depth less than two-thirds, while the other 55 cases with more advanced stromal invasion depth. As for the pathological differentiation grade, 21 cases were classified as well differentiation, 99 cases with moderate differentiation, and the other 13 cases with poor differentiation. In addition, 32 cases showed positive vagina invasion, 37 cases with positive parametrial invasion, 43 cases with lymphovascular invasion, and 55 cases with positive lymph node metastases. Among all cases, 72 patients were staged as FIGO stage I, while the other 61 cases were staged as FIGO stage II. The median DFS time of this cohort was 41 months, ranging 6–103 months.

**Table 1.** Correlations between MTL5 and clinicopathologic characteristics

Variables	Cases (n=133)	MTL5 protein level		P value
		Low (n=50)	High (n=83)	
Age (year)				0.407
< 45	62	21	41	
≥ 45	71	29	42	
Horizontal diffusion diameter				0.002*
< 4.0 cm	90	42	48	
≥ 4.0 cm	43	8	35	
Stromal invasion depth				0.089
< 2/3	78	34	44	
≥ 2/3	55	16	39	
Vagina invasion				0.214
Negative	101	35	66	
Positive	32	15	17	
Parametrial invasion				0.445
Negative	96	38	58	
Positive	37	12	25	
Lymphovascular invasion				0.111
Negative	90	38	52	
Positive	43	12	31	
Pathological differentiation				0.432
Well	21	8	13	
Moderate	99	35	64	
Poor	13	7	6	
Lymph node metastasis				0.005*
Negative	78	37	41	
Positive	55	13	42	
FIGO stage				<0.001*
Stage I	72	39	33	
Stage II	61	11	50	

* indicates P<0.05 with statistical significance.

### Expression of MTL5 in CESC

**Figure 1.**
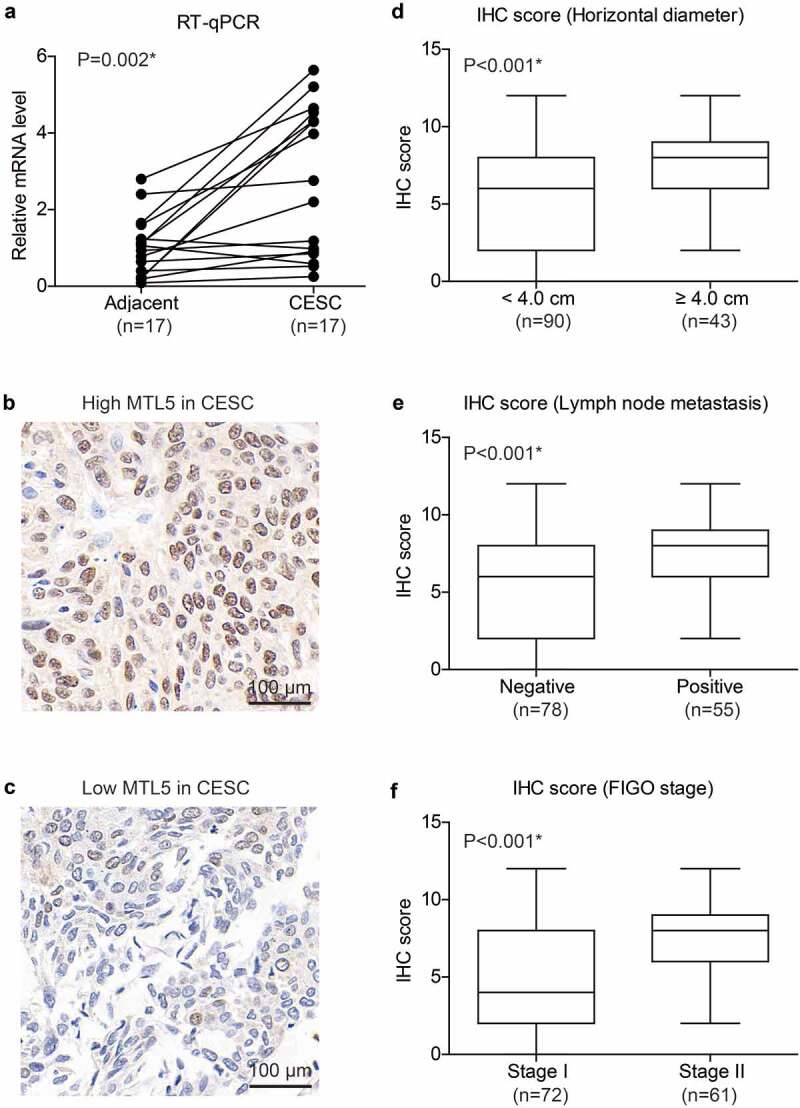
mRNA and protein expression of MTL5 in CESC.

We firstly tested the mRNA levels of MTL5 in the 17 fresh-frozen paired CESC tissues and adjacent nontumorous cervical tissues, which revealed a significantly higher MTL5-mRNA level in CESC tissues (Figure 1(a), p = 0.002). The distinct mRNA expression in CESC and tumor tissues engaged us to further investigate its protein expression pattern. According to the IHC data, MTL5 showed different protein expression levels in different CESC tissues (Figure 1(b-c)). After scoring the IHC results, we found that tumors with larger horizontal diffusion diameter exhibited higher MTL5 expression level (Figure 1(d), p < 0.001). Similarly, higher IHC scores were observed in patients with positive lymph node metastasis (Figure 1(e), p < 0.001) or advanced FIGO stage (Figure 1(f), p < 0.001). The correlations between high MTL5 expression and aggressive tumor characteristics indicated that MTL5 may participate in CESC progression.

(a) mRNA level of MTL5 was evaluated by RT-qPCR in 17 pairs of fresh-resected CESC tumor tissues and nontumorous ovarian tissues, indicating that CESCs possess higher MTL5-mRNA levels compared to normal cervical tissues. Data was analyzed by paired Student’s t-test (P = 0.002). (b) Representative high protein level of MTL5 in CESC tissues, showing predominant nucleus localization. (c) Representative low-protein immunostaining of MTL5 in CESC tissues. (d-f) Box plots showed the differential MTL5 protein expression in CESC tissues with different horizontal diameters (d), lymph node status (e), and FIGO stages (f). The Y-axis was presented as the IHC score of MTL5 in CESC tissues. Data was shown as mean ± SD and compared by unpaired Student’s t-test.

### Higher MTL5 is correlated with worse prognosis of CESC patients

**Figure 2.**
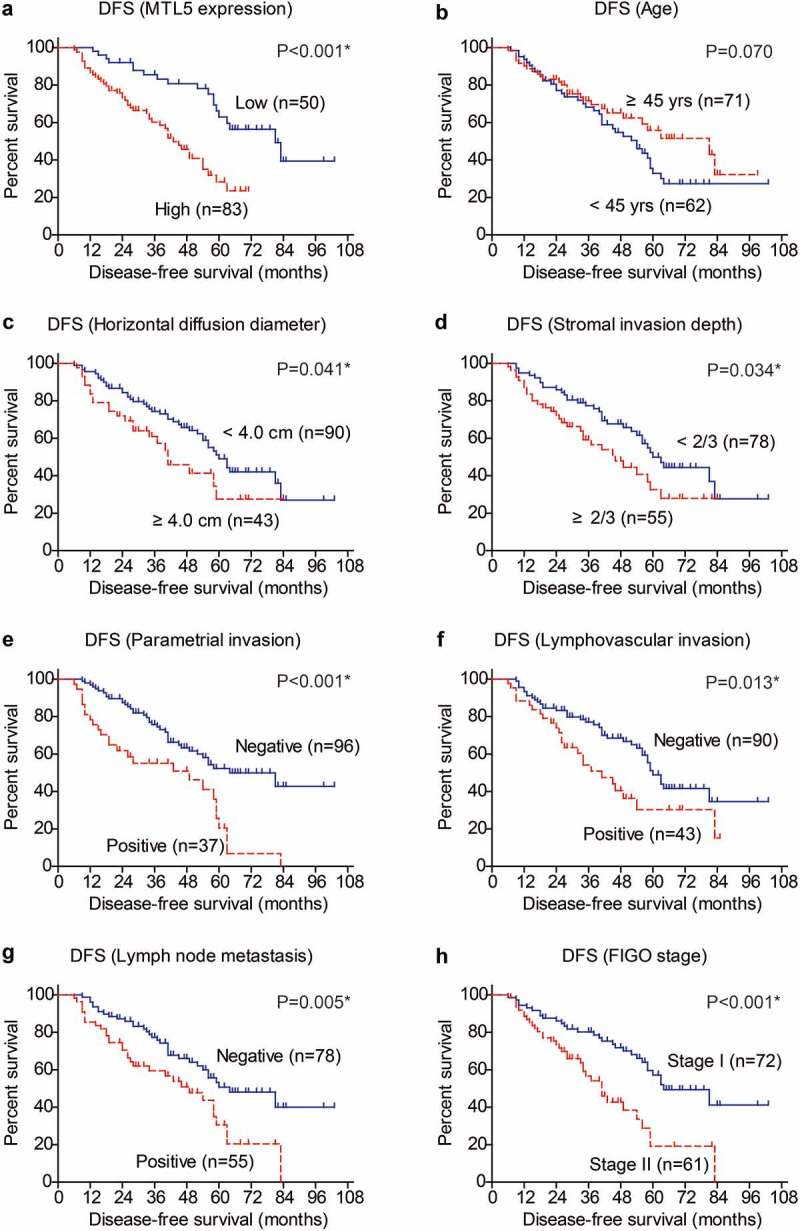
Disease-free survival analyses of CESC cohort.

The positive correlations between MTL5 protein level and unfavorable clinicopathological characteristics of CESC engaged us to further investigate whether MTL5 can help predict patients’ survival. To validate our hypothesis, we conducted Kaplan–Meier analyses and log-rank tests (Table 2). According to the IHC data, we divided patients into low-MTL5 group (n = 50) and high-MTL5 group (n = 83) as described in the Method section, which revealed that patients with higher MTL5 protein expression exhibited shorter DFS time (42.9 ± 2.6 months) compared to those with lower MTL5 levels (74.0 ± 4.9 months). The 5-year DFS rate was only 28.3% in high-MTL5 group, while was 63.0% in low-MTL5 group (Figure 2(a), p < 0.001). Interestingly, patients with younger age seems to have worse prognosis although the statistical difference was not significant (Figure 2(b), p = 0.070). As expected, patients with larger horizontal diffusion diameter (Figure 2(c), p = 0.041) or deeper stromal invasion depth (Figure 2(d), p = 0.034) were characterized with poorer DFS. The invasion parameters of CESC, including parametrial invasion (Figure 2(e), p < 0.001), lymphovascular invasion (Figure 2(f), p = 0.013), and lymph node metastasis (Figure 2(g), p = 0.005) all exhibited prognostic significance. Patients with FIGO stage II also showed unfavorable DFS compared to those with FIGO stage I (Figure 2(h), p < 0.001). According to our cohort, neither the vagina invasion nor pathological differentiation had statistically significant effect on patients’ DFS.

**Table 2.** Disease-free survival (DFS) analyses by Kaplan–Meier method and log-rank test

Variables	Cases (n=133)	5-year DFS rate (%)	Mean DFS (months)	P value
Age (year)				0.070
< 45	62	32.9%	56.0 ± 4.6	
≥ 45	71	55.9%	63.6 ± 4.8	
Horizontal diffusion diameter				0.041*
< 4.0 cm	90	49.0%	63.0 ± 4.1	
≥ 4.0 cm	43	27.6%	42.3 ± 4.9	
Stromal invasion depth				0.034*
< 2/3	78	49.9%	64.3 ± 4.4	
≥ 2/3	55	32.6%	47.4 ± 4.2	
Vagina invasion				0.357
Negative	101	42.5%	59.8 ± 3.8	
Positive	32	42.5%	53.4 ± 6.7	
Parametrial invasion				<0.001*
Negative	96	52.3%	68.0 ± 4.1	
Positive	37	20.5%	39.9 ± 4.3	
Lymphovascular invasion				0.013*
Negative	90	48.9%	64.8 ± 4.1	
Positive	43	30.3%	46.0 ± 4.8	
Pathological differentiation				0.273
Well	21	53.0%	60.4 ± 5.8	
Moderate	99	41.9%	58.5 ± 4.0	
Poor	13	25.2%	35.7 ± 4.9	
Lymph node metastasis				0.005*
Negative	78	50.7%	67.4 ± 4.5	
Positive	55	30.6%	45.7 ± 4.0	
FIGO stage				<0.001*
Stage I	72	57.2%	69.3 ± 4.5	
Stage II	61	19.2%	43.7 ± 3.8	
MTL5 expression				<0.001*
Low	50	63.0%	74.0 ± 4.9	
High	83	28.3%	42.9 ± 2.6	

* indicates P<0.05 with statistical significance.

Kaplan–Meier method was used to analyze the prognostic significances of retrieved clinicopathological characteristics, such as MTL5 protein level (a), age at diagnosis (b), horizontal diffusion diameter (c), stromal invasion depth (d), parametrial invasion (e), lymphovascular invasion (f), lymph node metastasis (g), and FIGO stage (h). Data was analyzed by log-rank test.

**Figure 3.**
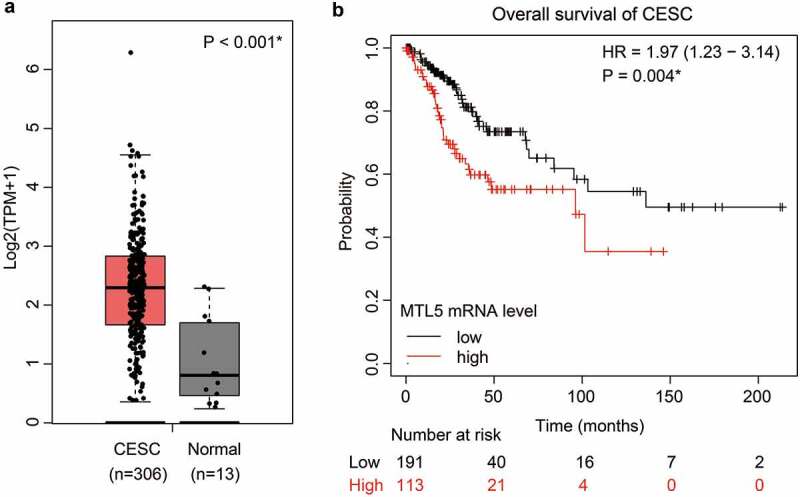
The mRNA level of MTL5 and its clinical significance in TCGA database.

Since our cohort only contains limited cases with early FIGO stages from a single medical center, we further searched the TCGA database to validate our findings. Consistent with our data, the mRNA transcription level of MTL5 was significantly lower in normal cervix than that in CESC tissues (Figure 3(a)). According to the TCGA database, the median overall survival time of CESC patients with lower MTL5-mRNA level was 136.2 months, while decreased to 96.3 months in those with higher MTL5-mRNA level (Figure 3(b), p = 0.004). Together with our data, the results implied that MTL5 may serve as a prognostic predictive factor for not only early-stage CESC patients, but also for all CESCs.

(a) The mRNA level of MTL5 in CESC tissues was extracted from TCGA database. By analyzing the transcripts per million (TPM) level of MTL5 in CESC tissues (n = 306) and normal cervix tissues (n = 13), we found that MTL5-mRNA level was significantly higher in CESC tissues (P < 0.001). (b) Relationship between MTL5-mRNA level and overall survival of CESC patients was plotted by Kaplan–Meier method, which indicated an unfavorable effect of MTL5 on CESC prognosis.

We next subjected the univariate variables whose P value was less than 0.1 into a multivariate Cox regression model (Table 3). As a result, both positive parametrial invasion (HR = 1.866, 95% CI 1.030–3.380, P = 0.040) and advanced FIGO stage (HR = 1.829, 95% CI 1.040–3.217, P = 0.036) showed independent unfavorable effect on CESC survival. Of note, higher MTL5 protein expression was also identified as a novel independent risk factor (HR = 1.961, 95% CI 1.034–3.720, P = 0.039). Therefore, our data provided initial evidence on the prognostic predictive role of MTL5 in CESC.

**Table 3.** Multivariate analysis by Cox regression model

Variables	Hazard ratio	95% CI	P value
Age	0.649	0.383-1.101	0.109
Horizontal diffusion diameter	1.028	0.578-1.827	0.926
Stromal invasion depth	1.378	0.814-2.334	0.232
Parametrial invasion	1.866	1.030-3.380	0.040*
Lymphovascular invasion	1.481	0.872-2.514	0.146
Lymph node metastasis	1.361	0.765-2.422	0.294
FIGO stage	1.829	1.040-3.217	0.036*
MTL5 expression	1.961	1.034-3.720	0.039*

* indicates P<0.05 with statistical significance.

### MTL5 promotes CESC proliferation in vitro and in vivo

**Figure 4.**
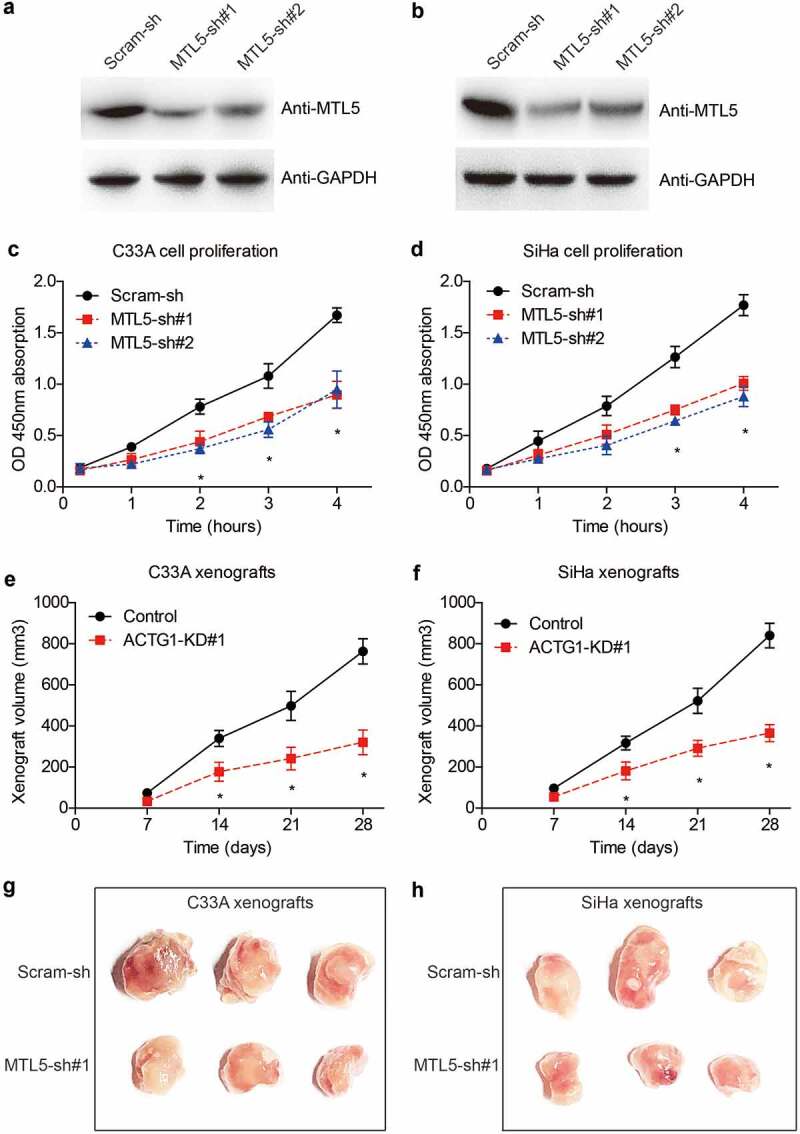
The effects of MTL5 on CESC proliferation and growth.

To provide more evidence on the tumor-related role of MTL5 in CESC, we silenced its expression in C33A and SiHa cell lines via shRNA transfection. After confirming the knockdown efficiencies (Figure 4(a-b)), transfected cells were subjected to in vitro proliferation test and in vivo xenograft experiments. As revealed by the CCK-8 assays, MTL5 interference led to a significant inhibition effect on the proliferation of C33A cells (Figure 4(c)). Similar results were observed in SiHa cells (Figure 4(d)).

(a, b) Two shRNAs targeting MTL5 (MTL5-sh#1 and MTL5-sh#2) as well as scrambled shRNA (Scram-sh) were transfected into C33A and SiHa cell lines, respectively. The knockdown efficiencies were tested by Western blotting. (c, d) CCK-8 assays reflected that MTL5-shRNA can significantly attenuate the proliferation of both C33A and SiHa cell lines. (e, f) The growth curves of xenografts generated by MTL5-sh#1 cells and Scram-sh cells showed that MTL5 interference remarkably impaired CESC tumor growth in vivo. (g, h) Images showed significantly different xenograft size after tumor isolation.

In addition, growth curves of in vivo xenografts demonstrated that MTL5-shRNA can sufficiently suppress CESC tumor growth (Figure 4(e-f)), which was validated by the size comparation after xenografts isolation (Figure 4(g-h)).

## Discussions

MTL5 refers to a 60 kDa protein that has cysteine-rich motifs and exerts metallothionein-like characteristics. Our knowledge regarding MTL5ʹs involvement in malignancies is limited. Cervical cancer represents one of the most important female genital cancers. CESC accounts for about 90% of all cervical malignancies and the prognosis are unsatisfied [17–19]. Here, we revealed that MTL5 expression was upregulated in CESC cells than that in nontumorous cervix tissues. Moreover, our data showed a positive correlation between MTL5 expression and tumor stage in a retrospective cohort that included 133 cases that underwent R0 surgical resection in our hospital. By analyzing the clinical outcomes, we found that high MTL5 was significantly correlated to an unfavorable disease-free survival of CESC. To validate our findings and minimize the regional bias, we extracted the TCGA data for further analyses. Consistent with its protein expression, higher MTL5-mRNA level also predicted poorer overall survival of CESC cases in the TCGA cohort. Therefore, our data proved that MTL5 exerts independent effect on the post-operative survival of CESC and can serve as an invaluable prognostic predictive factor.

The role of MTL5 on promoting cell proliferation was previously focused on its role in facilitating meiotic division in germ cells [20–23]. However, according to the data by Grzegrzolka et al., a positive correlation between MTL5 and Ki‑67 was observed in lung adenocarcinoma tissues, highlighting its potential role in cancer cell proliferation [11]. Their recent work showed that MTL5 interference indeed resulted in cell cycle arrest in lung cancer cells [24], indicating that MTL5 may also regulate the mitosis. Consistently, our data initially demonstrated the pro-proliferation role of MTL5 in CESC cells. According to our cellular results, silencing MTL5 led to a significant decrease on the proliferation capacity of CESC cells. Moreover, we validated the tumor-suppressing effect of MTL5-shRNA using xenograft mice models, providing the first in vivo evidence regarding its tumor-related function. The significant effects of MTL5-shRNAs on inhibiting CESC growth may also provide inspirations on developing novel targeted therapies.

There are several possible underlying mechanisms. On one hand, MTL5 may participate in transcription process by modulating the transcription factors considering its zinc-binding function [25]. On the other hand, MTL5 may positively regulate the expression of DNA replication licensing factors such as MCM5 and MCM7 in lung cancer [24]. However, the molecular mechanism downstream of MTL5 remains to be detailed in other malignancies. Future work may focus on mapping its subcellular transportation and localization during carcinogenesis to test its participation in the organization of chromatin. Besides, the single-nucleotide polymorphism (SNP) and splicing variants also deserve further investigation since MTL5-SNP had been reported to play a role in susceptibility to childhood B-cell acute lymphoblastic leukemia in Hispanics [26].

Our study has several limitations. Firstly, all the retrospectively enrolled cases were obtained from our hospital and may lead to regional or racial bias. We tried to make our major conclusion more convincible by retrieving the mRNA level of MTL5 from TCGA database. Secondly, our data only included patients who underwent R0 resection and was limited to FIGO stage I–II cases, therefore we did not illustrate the possible crosstalk between MTL5 with resistance of adjuvant therapies. Thirdly, this study focused more on exploring the clinically prognostic role of MTL5 in CESC, therefore we did not fully dig into its oncogenic signaling mechanisms. Instead, we validated our clinical results by assessing the effects of MTL5 on cell proliferation in two CESC cell lines as well as in mice xenografts. More in vitro and in vivo assays will be necessary to further demonstrating the detailed mechanism of MTL5 in tumor development and progression [27].

## Conclusions

MTL5 is upregulated in CESC than that in normal cervix tissues, whose higher expression is closely correlated with worse prognosis. Knockdown of MTL5 significantly attenuated CESC progression, highlighting that targeting MTL5 may serve as a novel direction for therapeutic development.

## Highlights

- MTL5 is aberrantly up-regulated in CESC tissues than that in normal cervix tissues.
- Higher MTL5 can help predict worse prognosis of CESC patients.
- MTL5 can positively regulate CESC growth both in vitro and in vivo.
